# A (GCC) repeat in SBF1 reveals a novel biological phenomenon in human and links to late onset neurocognitive disorder

**DOI:** 10.1038/s41598-022-19878-y

**Published:** 2022-09-14

**Authors:** Safoura Khamse, Samira Alizadeh, Stephan H. Bernhart, Hossein Afshar, Ahmad Delbari, Mina Ohadi

**Affiliations:** 1 Iranian Research Center on Aging, University of Social Welfare and Rehabilitation Sciences, Tehran, Iran; 2 IZBI, Interdisciplinary Centre for Bioinformatics, Universität Leipzig, Härtelstr. 16-18, 04107 Leipzig, Germany

**Keywords:** Evolution, Genetics, Molecular biology, Neuroscience, Diseases

## Abstract

The human *SBF1* (SET binding factor 1) gene, alternatively known as *MTMR5*, is predominantly expressed in the brain, and its epigenetic dysregulation is linked to late-onset neurocognitive disorders (NCDs), such as Alzheimer’s disease. This gene contains a (GCC)-repeat at the interval between + 1 and + 60 of the transcription start site (SBF1-202 ENST00000380817.8). We sequenced the *SBF1* (GCC)-repeat in a sample of 542 Iranian individuals, consisting of late-onset NCDs (N = 260) and controls (N = 282). While multiple alleles were detected at this locus, the 8 and 9 repeats were predominantly abundant, forming > 95% of the allele pool across the two groups. Among a number of anomalies, the allele distribution was significantly different in the NCD group versus controls (Fisher’s exact *p* = 0.006), primarily as a result of enrichment of the 8-repeat in the former. The genotype distribution departed from the Hardy–Weinberg principle in both groups (*p* < 0.001), and was significantly different between the two groups (Fisher’s exact *p* = 0.001). We detected significantly low frequency of the 8/9 genotype in both groups, higher frequency of this genotype in the NCD group, and reverse order of 8/8 versus 9/9 genotypes in the NCD group versus controls. Biased heterozygous/heterozygous ratios were also detected for the 6/8 versus 6/9 genotypes (in favor of 6/8) across the human samples studied (Fisher’s exact *p* = 0.0001). Bioinformatics studies revealed that the number of (GCC)-repeats may change the RNA secondary structure and interaction sites at least across human exon 1. This STR was specifically expanded beyond 2-repeats in primates. In conclusion, we report indication of a novel biological phenomenon, in which there is selection against certain heterozygous genotypes at a STR locus in human. We also report different allele and genotype distribution at this STR locus in late-onset NCD versus controls. In view of the location of this STR in the 5′ untranslated region, RNA/RNA or RNA/DNA heterodimer formation of the involved genotypes and alternative RNA processing and/or translation should be considered.

## Introduction

While of vast evolutionary and biological implications^1–8^, short tandem repeats (STRs) remain an underappreciated topic in comparison to single nucleotide substitutions^9,10^, partly because of their repetitive nature and hardship of accurate allele calling with the currently available methods.

Among various categories of STRs, CGG/GCC repeats are overrepresented in the exons of the human genome, and are mainly focused on because of their involvement in neurological disorders^11–14^. The human gene, *SBF1* (SET binding factor 1), also known as *MTMR5* (Myotubularin-related protein 5) contains an annotated (GCC)-repeat of 9-repeats in the 5′ untranslated region (UTR), between + 1 to + 60 of the transcription start site (TSS) (SBF1-202 ENST00000380817.8), which is in the top 1 percentile of (GCC)-repeats with respect to length^15^. *SBF1* is located at the extreme end of the long arm of chromosome 22 (22q13.33), and across all human tissues, reaches maximum expression in the cerebral cortex (https://www.proteinatlas.org/ENSG00000100241-SBF1/tissue). In comparison with other primate species, *SBF1* reaches maximum expression quantiles in the human brain and skeletal muscle (https://www.ncbi.nlm.nih.gov/IEB/Research/Acembly)16. In line with the above, aberrant regulation of the gene networks in which *SBF1* plays a role has been reported in late-onset neurocognitive disorders (NCDs), such as Alzheimer’s disease (AD)^17^.

Here we sequenced the *SBF1* (GCC)-repeat in a sample of humans, consisting of late-onset NCDs and controls, and performed structural and accessibility analysis of exon 1 (encompassing this repeat) with various (GCC) repeats. We also studied the status of this (GCC)-repeat across vertebrates.

## Materials and methods

### Subjects

Five hundred forty-two unrelated Iranian subjects of ≥ 60 years of age, consisting of late-onset NCD patients (DSM-5) (N = 260) and controls (N = 282) were recruited from the provinces of Tehran, Qazvin, and Rasht. In each NCD case, the Persian version of the Abbreviated Mental Test Score (AMTS)^18,19^ was implemented (AMTS < 7 was an inclusion criterion for NCD), medical records were reviewed in all participants, and CT-scans were taken where possible. Furthermore, in a number of subjects, the Mini-Mental State Exam (MMSE) Test^20^ was implemented in addition to the AMTS. A score of < 24 was an inclusion criterion for NCD. The Persian version of the AMTS is a valid cognitive assessment tool for older Iranian adults, and can be used for NCD screening in Iran^18^. The onset of neurocognitive impairment was also investigated by clinical interviews, which confirmed the occurrence of those symptoms at ≥ 60 years. The control group was selected based on cognitive AMTS of > 7 and MMSE > 24, lack of major medical history, and normal CT-scan where possible. The cases and controls were matched based on age, gender, and residential district. The subjects' informed consent was obtained (from their guardians where necessary) and their identities remained confidential throughout the study. The research was approved by the Ethics Committee of the Social Welfare and Rehabilitation Sciences, Tehran, Iran, and was consistent with the principles outlined in an internationally recognized standard for the ethical conduct of human research. All methods were performed in accordance with the relevant guidelines and regulations.

### Allele and genotype analysis of the *SBF1* (GCC)-repeat

Genomic DNA was obtained from peripheral blood using a standard salting out method. PCR reactions for the amplification of the *SBF1* (GGC)-repeat were set up with the following primers:

- **Forward**: TCTGGACCAATGGAGATGCG
- **Reverse**: GAAGTAGTCCGCGAGCCG

PCR reactions were carried out in a final volume of 20 µl, at a final concentration of 30% high-GC buffer, in a thermocycler (Peqlab-PEQStar) under the following conditions: initial denaturation at 95 °C for 5 min, 40 cycles of denaturation at 95 °C for 45 s, annealing at 55 °C for 45 s, and extension at 72 °C for 1 min, and a final extension at 72 °C for 10 min. All samples included in this study were sequenced by the forward primer, using an ABI 3130 DNA sequencer (Suppl. 1).

### Statistical analysis

The SPSS Fisher’s exact test was used to compare allele and genotype distribution between NCD and control groups. Fisher’s exact test was also used for the 6/8 versus 6/9 genotypes. The Hardy–Weinberg principle (HWP) was tested using the exact test of Hardy–Weinberg proportion for multiple alleles^21^.

### Structural analysis of the human *SBF1* with different numbers of (GCC)-repeats

We investigated accessibility i.e., probability of being unpaired, of exon 1 of the human *SBF1* gene, with 5 to 10 (GCC)-repeats, using the accessibility computation of the ViennaRNA package (RNAplfold with -W 300 -L 300 -u 10)^22,23^. We compared the accessibilities of all regions of 10 nt length. Furthermore, we used RNAup -b^24^ to compare possible interactions in homodimeric and heterodimeric *SBF1* first exon with different numbers of (GCC)-repeats.

### Analysis of the *SBF1* (GCC)-repeat across vertebrates

The interval between + 1 and + 100 of the TSS of the *SBF1* was searched across all species in which *SBF1* was annotated, based on Ensembl 104. The Ensembl alignment program was used for the sequence alignments across the selected species.

## Results

### The ***SBF1*** (GCC)-repeat allele distribution was significantly different in the NCD group versus controls

**Figure 1:**
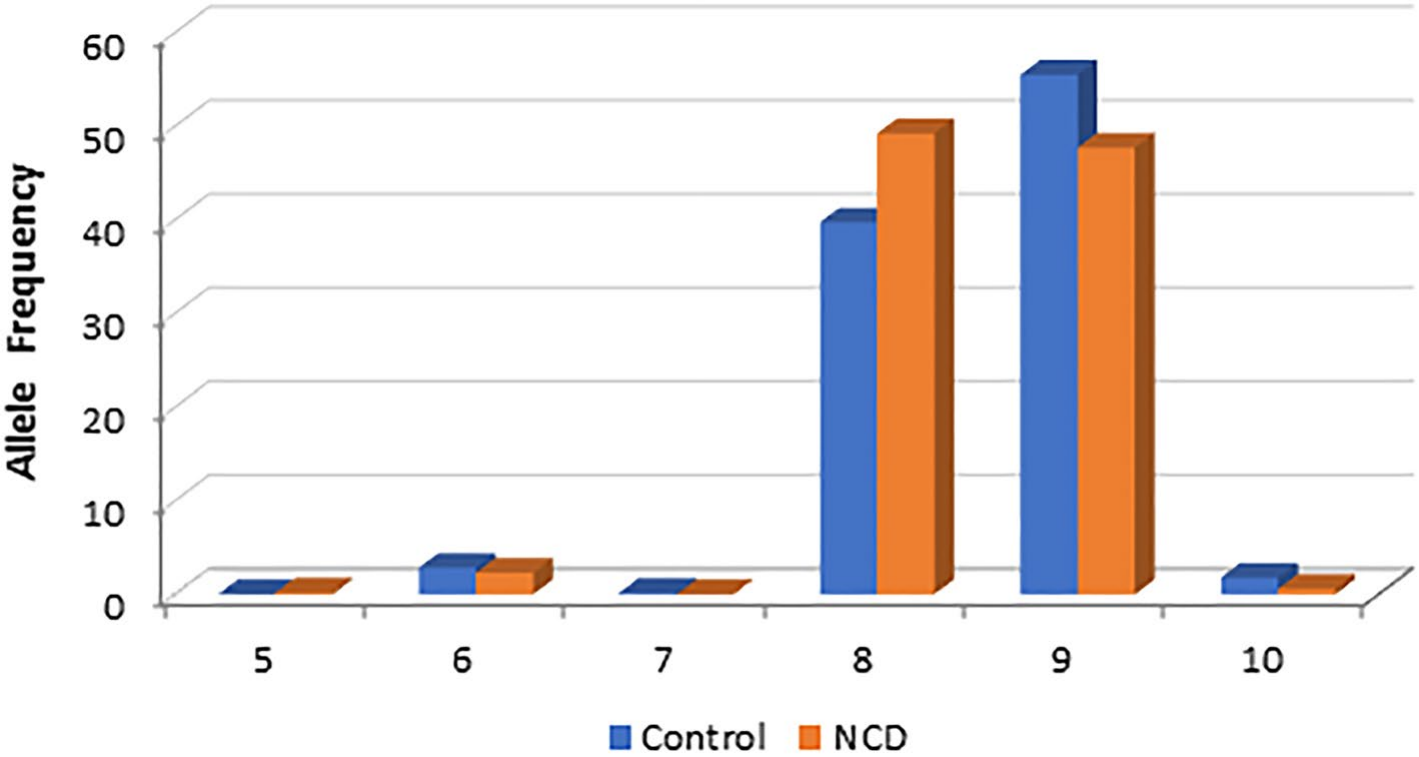
Allele frequency of the *SBF1* (GCC)-repeat in the human samples studied. While multiple alleles were detected, the 8 and 9-repeat alleles were predominantly abundant. Significant excess of the 8-repeat was detected in the NCD group versus controls.

We detected two predominantly abundant alleles of 8 and 9-repeats, which formed > 95% of the allele pool across the two groups (Table 1, Fig. 1). At significantly lower frequencies, we detected repeats of 5, 6, 7, and 10, with frequencies of < 0.03. The allele frequency distribution was significantly different in the NCD group versus controls (Fisher’s exact *p* = 0.006). Specifically, the frequency ratio of the 8 and 9 repeats was in the reverse order in the NCD group as a result of excess of the 8-repeat in this group.

**Table 1:** Allele distribution of the human *SBF1* (GCC) repeat in the NCD and control groups.

Alleles * Group Crosstabulation			
	Groups^a^		Total
	Controls	NCDs	
**Alleles**			
5-repeat			
Count	0	1	1
%	0.0%	0.2%	0.1%
6-repeat			
Count	16	12	28
%	2.8%	2.3%	2.6%
7-repeat			
Count	1	0	1
%	0.2%	0.0%	0.1%
8-repeat			
Count	224	256	480
%	39.7%	49.2%	44.3%
9-repeat			
Count	313	248	561
%	55.5%	47.7%	51.8%
10-repeat			
Count	10	3	13
%	1.8%	0.6%	1.2%
**Total**			
Count	564	520	1084
%	100.0%	100.0%	100.0%

^a^Fisher’s exact *p* = 0.006. Counts and % represent within each group.

### The *SBF1* (GCC)-repeat genotype distribution deviated from HWP in both groups and was different between the two groups

**Figure 2:**
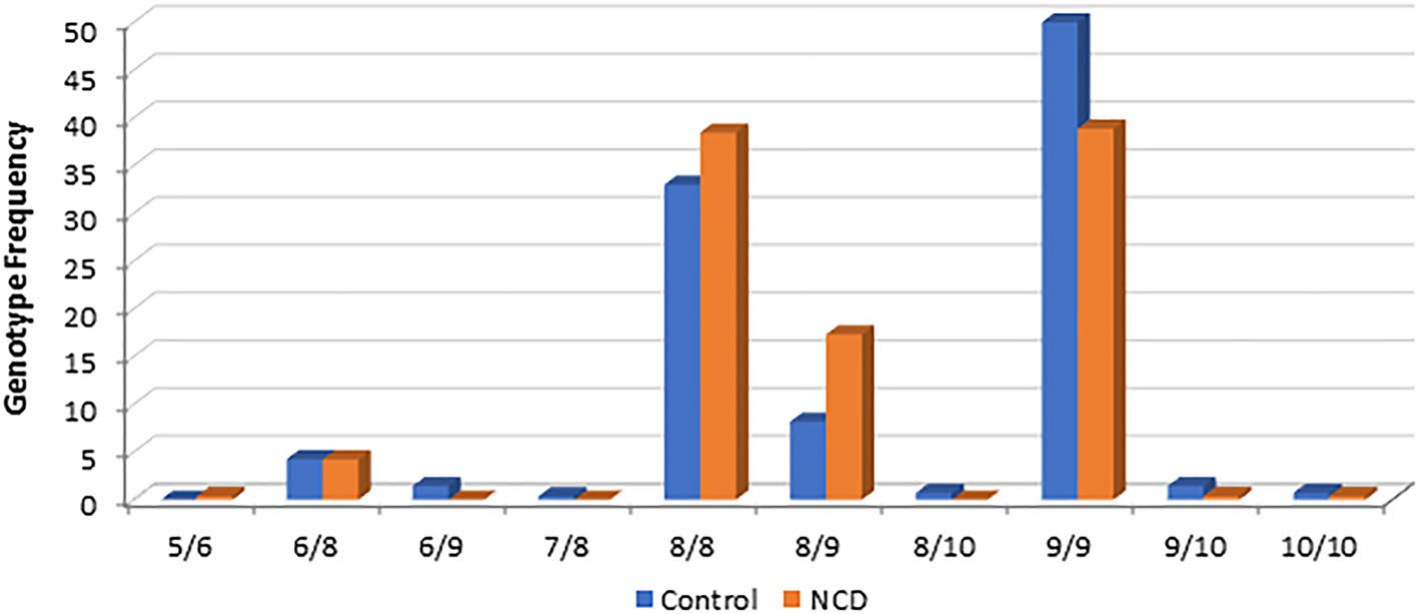
Genotype frequency of the *SBF1* (GCC)-repeat in the human samples studied. The genotype distribution departed from HWP in both groups and was different between the two groups.

The genotype distribution was anomalous in both NCD and control groups, and deviated from the HWP (*p* < 0.001). Specifically, rather than an expected > 45% 8/9 genotype based on the 8 and 9-repeat allele frequencies, we detected < 18% of that genotype across the two groups (Table 2, Fig. 2). There were other discrepancies in the genotype distribution The 6/8 genotype was significantly more detected than the 6/9 genotype across the human samples studied (Fisher’s exact *p* = 0.0001).

**Table 2:** Genotype distribution of the human *SBF1* (GCC) repeat in the NCD and control groups.

Genotypes * Group Crosstabulation			
	Groups^a^		Total
	Controls	NCDs	
**Genotypes**			
5/6			
Count	0	1	1
%	0.0%	0.4%	0.2%
6/8			
Count	12	11	23
%	4.3%	4.2%	4.2%
6/9			
Count	4	0	4
%	1.4%	0.0%	0.7%
7/8			
Count	1	0	1
%	0.4%	0.0%	0.2%
8/8			
Count	93	100	193
%	33.0%	38.5%	35.6%
8/9			
Count	23	45	68
%	8.2%	17.3%	12.5%
8/10			
Count	2	0	2
%	0.7%	0.0%	0.4%
9/9			
Count	141	101	242
%	50.0%	38.8%	44.6%
9/10			
Count	4	1	5
%	1.4%	0.4%	0.9%
10/10			
Count	2	1	3
%	0.7%	0.4%	0.6%
**Total**			
Count	282	260	542
%	100.0%	100.0%	100.0%

^a^Fisher’s exact *p* = 0.001. Counts and % represent within each group.

The genotype distribution was significantly different between the NCD and control groups (Fisher’s exact *p* = 0.001) (Table 2), Specifically, we detected significant enrichment of the 8/9 genotype in the NCD group versus controls, and reverse ratio of 8/8 and 9/9 genotypes between the two groups.

### Identification of an extreme genotype in the NCD group only

**Figure 3:**
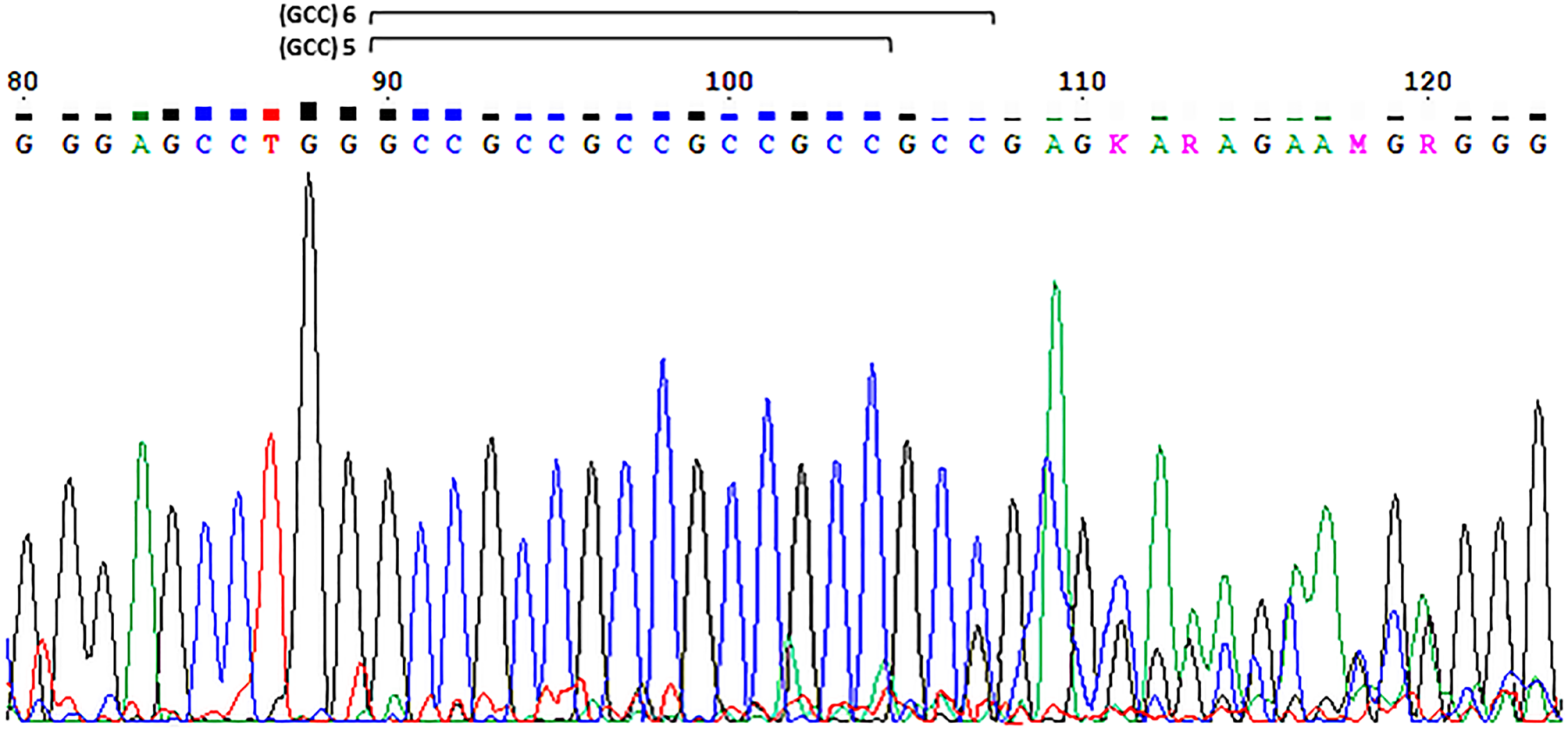
Identification of a genotype at the short extreme of the allele range in one instance of late-onset NCD.

We detected a genotype at the extreme short end of the allele range in one instance of late-onset NCD. This genotype was 5/6 (Fig. 3), and was detected in an 85-year-old female case of NCD with AMTS = 3, and suspected of having late-onset AD. The shortest allele detected in the control group was 6-repeats, and 5-repeats was not detected in this group.

### The number of (GCC)-repeats may change the RNA secondary structure and interaction sites

**Figure 4:**
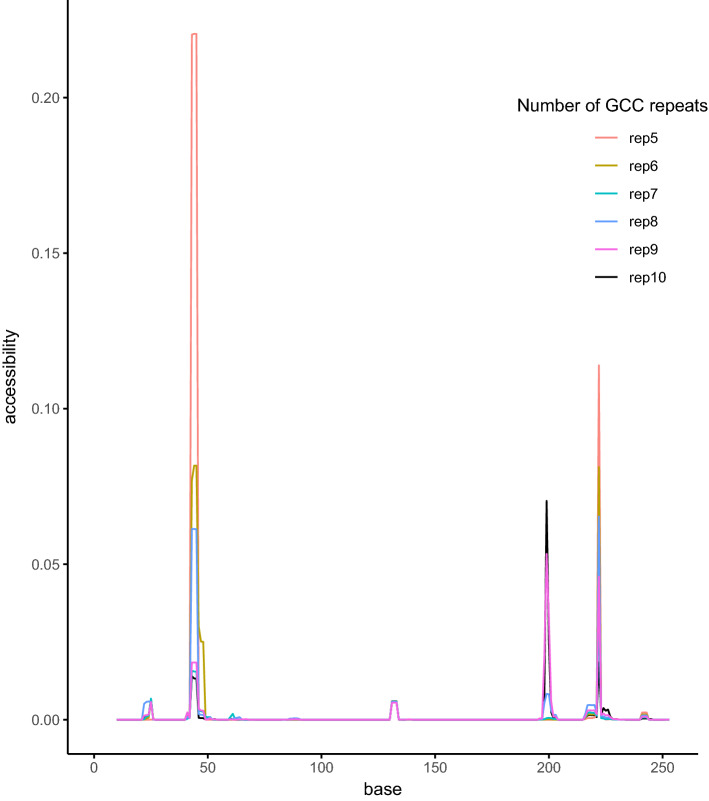
Accessibility (probability of being unpaired) of all regions of 10 nt length, ending at base x for the first exon of human *SBF1* with 5 to 10-repeats. Differences in 3 regions were detected, at about nt 50, about nt 200, and about nt 220.

The accessibility of exon 1 of human *SBF1* varied with the number of (GCC)-repeats in three regions, around nucleotide (nt) 50 (at the (GCC)-repeat itself), at about nt 200 (at the translation start site) and at nt 220 (all nt relative to the TSS based on Ensembl transcript ID: ENST00000380817.8 SBF1-202) (Fig. 4). Furthermore, we analyzed where the preferred interaction sites would be, and found that there are two different groups of interaction sites (Table 3): in one group, the best molecular interaction occurs between nt 119–130 and nt 219–230, while the other group has interactions between nt 182–200 and nt 193–211.

**Table 3:** Interaction groups across various human *SBF1* (GCC)-repeats^a^.

Group 1	((((((.(((((&))))).)))))) CGUGCUGGUGGC&GCCAUGAGCGCG 5 vs 5, 5 vs 6, 5 vs 7, 5 vs 8, 5 vs 9, 5 vs 10, 6 vs 6, 6 vs 7, 6 vs 8, 8 vs 8
Group 2	(((((((((.((..(((((&)))))..)).))))))))) GCCAUGGCGCGGCUCGCGG&CCGCGUCCCUCGCCAUGGC 6 vs 9, 6 vs 10, 7 vs 7, 7 vs 8, 7 vs 9, 7 vs 10, 8 vs 9, 8 vs 10, 9 vs 9, 9 vs 10, 10 vs 10

^a^Lengths with interaction structure and sequences in bracket-dot notation (matching parenthesis are opening and closing bases of a base pair, dots are unpaired bases, and separate the two interacting sequences).

### *SBF1* (GGC)-repeat expanded specifically in primates

**Figure 5:**
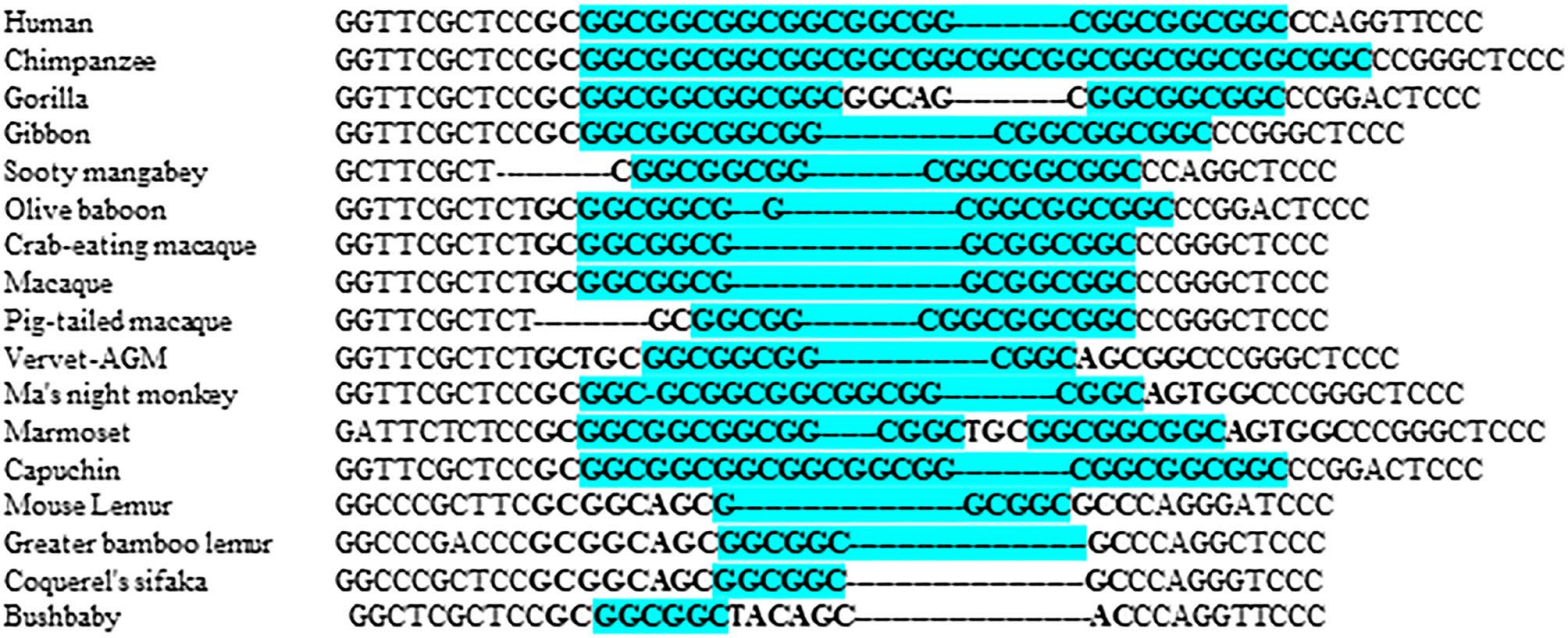
Sequence alignment of the *SBF1* (GCC)-repeat across selected vertebrate species. The (GCC)-repeat expanded beyond 2-repeats in primates.

Across all the vertebrate species studied, the *SBF1* (GCC)-repeat specifically expanded beyond 2-repeats in primates (Fig. 5).

## Discussion

The primary importance of (GCC)-repeats stems from a possible link between that type of STR and natural selection, mainly for two reasons: Firstly, (GCC)-repeats are specifically enriched in the exons. Secondly, GC-rich sequences are mutation hotspots^25^, and frequently interrupted by single nucleotide substitutions. The intact occurrence of the *SBF1* (GCC)-repeat in primates, and not in any other order, supports selective advantage in this order.

In both NCD and control groups, the genotype distribution significantly departed from HWP. Not only the expected heterozygosity for the observed allele frequencies was dramatically compromised, but also certain heterozygous/heterozygous ratios were biased.

The accumulated homozygosity could not be attributed to the excess of consanguineous marriages in Iran, as excess of homozygosity in consanguineous societies can contribute to between 2 and 11% homozygosity at a given locus^26,27^. Sampling error is another explanation for the observed genotypes. All samples were collected from the same districts in Iran, and the results were replicated in both groups. Rare primer binding site mutations are known to provoke null alleles in STRs, and lead to false homozygous genotypes^28–30^. In a review by Dakin and Avise, it was reported that whereas null alleles in frequencies typically reported in the literature introduce rather inconsequential biases on average exclusion probabilities, they can introduce substantial errors into empirical assessments of specific mating events by leading to high frequencies of false parentage exclusions^31^. While the scope of our research was not assessing specific matings, we double-checked 70 random samples across the two groups with alternative primers (Forward: TCAGGGCTTGACGACAGC, Reverse: CTCGACCCTCAGACCCAG), with alternative binding sites to the original primers, and identical PCR conditions to the original primer set, which confirmed our initial genotyping results. It should be noted that this preliminary study needs to be replicated with independent samples by other groups, in order to confirm the results.

A likely hypothesis that may be put forward is that certain heterozygous genotypes might have been selected against in human in the process of evolution. The studied (GCC)-repeat is located in the 5′ UTR, and it may be speculated that the heterodimer RNAs of, for example, 8/9 and 6/9 have a detrimental effect on the downstream events, such as transcript processing and translation. A possible mechanism might be connected to RNA structure and accessibility. Experimental synthetic stem-loop RNAs have been reported to alter the expression of a number of genes in bacteria^32^. We could show that the accessibility changes with the number of (GCC)-repeats, and can affect at least exon 1. For example, the 6/8 and 6/9 RNA interactions were differentially grouped in groups 1 and 2, respectively (Table 3).

*SBF1* is predominantly expressed in the brain and skeletal muscle, and the protein encoded by this gene is a member of the myotubularin family. Myotubularin-related proteins, namely MTMR2, MTMR13/SBF2 and MTMR5/SBF1 are mainly involved in regulating endolysosomal trafficking^33^ and mitochondrial functioning^34^. Dysregulation of *SBF1* is linked to late-onset NCDs such as AD^17^, which is also indicated by the observed genotype anomalies in the NCD group versus controls in our study. An isolate instance of an NCD patient harboring a genotype that consisted of extreme short alleles, may be of significance, while random co-occurrence should also be considered as a possibility. The secondary structure and accessibility effect of the 5/6 genotype were dramatically divergent, and the 5-repeat allele length was not detected in the control group. It is possible that low frequency alleles at the extreme ends of the allele distribution curve are subject to negative natural selection^8,12,35^.

It remains to be clarified how certain heterozygous genotypes might have been selected against at this locus in human. It is also warranted that this STR is sequenced in larger samples and in a spectrum of neurological disorders.

## Conclusion

We report indication of a novel biological phenomenon, in which there is significant selection against certain heterozygous genotypes at a STR locus in the human population. We also report different allele and genotype distribution in late-onset NCD versus controls at this locus. In view of the location of the (GCC)-repeat in the 5′ UTR of the *SBF1* gene, it is speculated that specific RNA/RNA or DNA/RNA heterodimers may exert effects that are selected against in the course of evolution. It should be noted that this is a pilot study, which needs to be replicated by independent groups and in different samples.

## Supplementary Information

Supplementary Information.

## Data Availability

The datasets used and analyzed during the current study are included in this published article (Suppl. 1).

## Abbreviations

AMTS: Abbreviated Mental Test Score
HWP: Hardy–Weinberg principle
MTMR5: Myotubularin-Related Protein 5
nt: Nucleotide
SBF1: SET binding factor 1
STR: Short tandem repeat
TSS: Transcription start site
UTR: Untranslated region

## References

[1] Nikkhah M, Rezazadeh M, Khorram Khorshid HR, Biglarian A, Ohadi M (2016). An exceptionally long CA-repeat in the core promoter of SCGB2B2 links with the evolution of apes and Old World monkeys. Gene.

[2] Mohammadparast S, Bayat H, Biglarian A, Ohadi M (2014). Exceptional expansion and conservation of a CT-repeat complex in the core promoter of PAXBP1 in primates. Am J Primatol..

[3] Afshar H (2020). Evolving evidence on a link between the ZMYM3 exceptionally long GA-STR and human cognition. Sci. Rep..

[4] Watts P (2017). Stabilizing selection on microsatellite allele length at arginine vasopressin 1a receptor and oxytocin receptor loci. Proc. R. Soc. B Biol. Sci..

[5] Hannan AJ (2018). Tandem repeats mediating genetic plasticity in health and disease. Nat. Rev. Genet..

[6] Ranathunge C (2021). Microsatellites as agents of adaptive change: An RNA-Seq-based comparative study of transcriptomes from five helianthus species. Symmetry..

[7] Fotsing SF (2019). The impact of short tandem repeat variation on gene expression. Nat Genet..

[8] Afshar H (2020). Natural selection at the NHLH2 core promoter exceptionally long CA-repeat in human and disease-only genotypes in late-onset neurocognitive disorder. Gerontology.

[9] Press MO, Hall AN, Morton EA, Queitsch C (2019). Substitutions are boring: some arguments about parallel mutations and high mutation rates. Trends Genetics.

[10] Press MO, Carlson KD, Queitsch C (2014). The overdue promise of short tandem repeat variation for heritability. Trends Genet..

[11] Annear DJ (2021). Abundancy of polymorphic CGG repeats in the human genome suggest a broad involvement in neurological disease. Sci. Rep..

[12] Jafarian Z (2021). Natural selection at the RASGEF1C (GGC) repeat in human and divergent genotypes in late-onset neurocognitive disorder. Sci. Rep..

[13] Khamse S (2021). Novel implications of a strictly monomorphic (GCC) repeat in the human PRKACB gene. Sci. Rep..

[14] Tang H (2017). Profiling of short-tandem-repeat disease alleles in 12,632 human whole genomes. Am J Hum Genet..

[15] Namdar P (2015). Exceptionally long 5′ UTR short tandem repeats specifically linked to primates. Gene.

[16] Thierry-Mieg D, Thierry-Mieg J (2006). AceView: A comprehensive cDNA-supported gene and transcripts annotation. Genome Biol..

[17] Li P (2019). Epigenetic dysregulation of enhancers in neurons is associated with Alzheimer's disease pathology and cognitive symptoms. Nat. Commun..

[18] Foroughan M (2017). Validity and reliability of abbreviated mental test score (AMTS) among older Iranian. Psychogeriatrics.

[19] Hodkinson HM (1972). Evaluation of a mental test score for assessment of mental impairment in the elderly. Age Ageing.

[20] Carpenter CR (2019). Accuracy of dementia screening instruments in emergency medicine: A diagnostic meta-analysis. Acad Emerg Med..

[21] Guo SW, Thompson EA (1992). Performing the exact test of Hardy-Weinberg proportion for multiple alleles. Biometrics.

[22] Bernhart SH, Mückstein U, Hofacker IL (2011). RNA accessibility in cubic time. Algorithms Mol. Biol..

[23] Lorenz R (2011). ViennaRNA package 2.0. Algorithms Mol. Biol..

[24] Mückstein U (2006). Thermodynamics of RNA–RNA binding. Bioinformatics.

[25] Nesta AV, Tafur D, Beck CR (2021). Hotspots of human mutation. Trends Genet..

[26] Woods CG (2006). Quantification of homozygosity in consanguineous individuals with autosomal recessive disease. Am. J. Hum. Genet..

[27] Kirin M (2010). Genomic runs of homozygosity record population history and consanguinity. PLoS ONE.

[28] Blais J (2015). Risk of misdiagnosis due to allele dropout and false-positive PCR artifacts in molecular diagnostics: Analysis of 30,769 genotypes. J Mol Diagn..

[29] Yao Y (2018). Null alleles and sequence variations at primer binding sites of STR loci within multiplex typing systems. Leg. Med. (Tokyo).

[30] Li F (2014). Identification of new primer binding site mutations at TH01 and D13S317 loci and determination of their corresponding STR alleles by allele-specific PCR. Forensic Sci. Int. Genet..

[31] Dakin EE, Avise JC (2004). Microsatellite null alleles in parentage analysis. Heredity.

[32] Paulus M, Haslbeck M, Watzele M (2004). RNA stem-loop enhanced expression of previously non-expressible genes. Nucleic Acids Res..

[33] Mammel AE (2022). Distinct roles for the Charcot-Marie-Tooth disease-causing endosomal regulators Mtmr5 and Mtmr13 in axon radial sorting and Schwann cell myelination. Hum. Mol. Genet..

[34] Berti B (2021). Bi-allelic variants in MTMR5/SBF1 cause Charcot-Marie-Tooth type 4B3 featuring mitochondrial dysfunction. BMC Med. Genomics.

[35] Khamse S (2022). Predominant monomorphism of the RIT2 and GPM6B exceptionally long GA blocks in human and enriched divergent alleles in the disease compartment. Genetica.

